# Pigeons (*Columba livia domestica*) Are Susceptible to Infectious Bursal Disease: A Comparative Study of Their Hematological and Serum Biochemical Alterations

**DOI:** 10.3389/fvets.2021.673398

**Published:** 2021-06-04

**Authors:** Ochuko Orakpoghenor, Sunday Blessing Oladele, Paul Ayuba Abdu, Talatu Patience Markus, Samson James Enam, Aliyu Danlami Andamin, Muhammed Shuaib Muhammed, Suleman Geidam Usman, King Akpofure Nelson Esievo

**Affiliations:** ^1^Veterinary Pathology, Ahmadu Bello University, Zaria, Nigeria; ^2^Veterinary Medicine, Ahmadu Bello University, Zaria, Nigeria; ^3^Veterinary Microbiology, Ahmadu Bello University, Zaria, Nigeria

**Keywords:** infectious bursal disease, pigeons, sentinel, hematology, serum biochemistry

## Abstract

The hematological and serum biochemical alterations following very virulent infectious bursal disease virus (IBDV) infection in pigeons and chickens were evaluated in this study. Sixty IBDV seronegative birds comprising 30 (3–6 weeks old) pigeons and 30 (3 weeks old) chickens were randomly divided as follows: 10 uninoculated pigeons only, 10 inoculated pigeons + 10 sentinel chickens, 10 inoculated chickens + 10 sentinel pigeons, and 10 uninoculated chickens. Inoculated birds were administered 0.20 ml of inoculum containing very virulent IBDV (vvIBDV). Blood was collected postinoculation/exposure (pi/pe) and processed for hematology and biochemistry. The results revealed significantly (*P* < 0.05) increased packed cell volume, decreased mean corpuscular hemoglobin (MCH), and MCH concentration (MCHC) in inoculated and sentinel chickens. Total leukocyte count (TLC), heterophil, and heterophil/lymphocyte (H/L) ratio were increased from 3 dpi/dpe in inoculated pigeons and from 3 to 14 dpi/dpe in inoculated and sentinel chickens. At 10 and 14 dpi/dpe, there was significantly (*P* < 0.05) increased serum total protein and globulin concentrations and decreased albumin/globulin ratio in pigeons and chickens. Serum urea concentration showed significant (*P* < 0.05) increase in inoculated and sentinel chickens at 3 and 4 dpi/dpe. To the authors' knowledge, this is the first report on hematological and serum biochemical alterations due to vvIBDV infection in pigeons.

## Introduction

Infectious bursal disease (IBD) or Gumboro disease (GD) caused by infectious bursal disease virus (IBDV) is characterized by inflammation of the bursa, bursal atrophy, and immunosuppression in chickens between 3 weeks and 3 months of age (1–3). The virus responsible for IBD possesses selective tropism for lymphoid tissue with the bursa of Fabricius (BF) being the primary target organ where it affects immature B lymphocytes (4, 5). Other lymphoid cells in the bone marrow, thymus, spleen, Peyer's patches, cecal tonsils, and Harderian glands have been reported to be also affected by IBDV (6). Chickens and turkeys constitute the natural host of IBDV, and transmission is primarily via fecal–oral route. Two serotypes of IBDV (1 and 2) have been identified, with serotype 1 considered to be pathogenic to chickens and serotype 2 considered avirulent (7, 8). Based on antigenicity and virulence, serotype 1 has been further classified into classical virulent, attenuated, antigenic variant, and very virulent IBDV (5, 9). In free-living wild birds, IBDV has been reported to induce lymphoid depletion with subclinical infection (10). In addition, serological evidence of serotype 1 IBDV infection in free-living wild birds suggests their possible susceptibility and role in the epidemiology of IBDV (7, 11).

Studies have documented hematological alterations in chickens, turkeys, and ducks due to IBDV with no information on the hematological alterations in domestic pigeons (*Columba livia domestica*) (12, 13). Pigeons have close interactions with chickens at backyard and on commercial poultry farms as well as live bird markets. In addition, there is an increase in pigeon production for consumption as meat and for research needs. Information on their susceptibility to various poultry diseases such as IBD would provide the impetus for enhanced production. Hence, in this study, the hematological and serum biochemical alterations following very virulent infectious bursal disease virus (IBDV) infection in pigeons (*C. livia domestica*) and chickens were evaluated.

## Materials and Methods

### Ethical Approval

The ethics governing the use and conduct of experiments on animals were strictly observed, and the experimental protocol was approved by the Ahmadu Bello University Committee on Animal Use and Care (ABUCAUC) with approval number ABUCAUC/2021/002.

### Experimental Birds

Thirty-day-old Institut de Sélection Animale (ISA) white cockerels were acquired from a reputable hatchery. When the chickens were 3 weeks old, they were used for the experiment. In addition, thirty 3–8-week-old domestic pigeons were acquired from local breeders and used for the experiment.

### Experimental Design

The pigeons were divided into three groups (A–C) comprising 10 birds each. In addition, the chickens were divided into three groups (I–III) comprising 10 birds each. Pigeons in group A (uninoculated pigeons) and chickens in group I (uninoculated chickens) were not inoculated with vvIBDV and kept separately; pigeons in group B (inoculated pigeons) and chickens in group II (inoculated chickens) were inoculated with Nigerian strain of vvIBDV orally (10^9.76^ CID_50_/ml). Pigeons in group C (sentinel pigeons) and chickens in group III (sentinel chickens) were uninoculated and kept together with inoculated chickens and inoculated pigeons, respectively. Hence, the four isolated groups of birds used for the experiment comprised of the following: 10 uninoculated pigeons only, 10 uninoculated chickens only, 10 inoculated pigeons + 10 sentinel chickens, and 10 inoculated chickens + sentinel pigeons. Birds in each group were kept in four separate pens and provided feed and water *ad libitum*. In addition, the surroundings of each pen were continuously fumigated.

### Blood Sample Collection

Prior to inoculation with vvIBDV, blood samples were collected from all pigeons and chickens via brachial veins, and the sera were harvested. The sera were confirmed seronegative for IBDV antibody using enzyme-linked immunosorbent assay (ELISA).

Blood was also collected from pigeons and chickens on days 0, 1, 2, 3, 4, 5, 6, 7, 10, and 14 postinoculation and/or exposure (dpi/dpe) and divided into two parts. One part was emptied into a labeled tube containing ethylenediaminetetraacetic acid (EDTA) as anticoagulant for hematological analyses. The other part of blood was poured into a labeled plain tube to harvest serum and used for serum biochemical analyses and IBDV antibody detection using ELISA (ID Screen^®^ IBD VP2, ID.vet, Innovative Diagnostics, Grabels, France).

### Hematological Analyses

The packed cell volume (PCV) was determined using a standard technique (14). This involved filling up of a non-heparinized capillary tube with blood to about three-quarters of its length from one end and sealing the other end and centrifugating it at 4,383 × g for 5 min using a Saitexiangyi TG12MX^®^ Microhematocrit centrifuge machine (Hunan Saite xiangyi centrifuge instrument Co., Ltd., Changsha, China). After that, the PCV value was measured using the Hawksley^®^ Microhematocrit Reader (Hawksley, Lancing, UK) and recorded as percentage of blood volume.

The Natt–Herrick solution (1:200 dilution) and improved Neubauer hemocytometer (15) were used to determine total leukocyte (TLC) or white blood cell (TWBC) counts. The cells were viewed using a light microscope (Olympus XSZ-107BN, Wincom Company Limited, Changsha, China) at a low power magnification (×40) and counted by means of a tally counter. Both sides of the hemocytometer were counted, and the average was calculated.

Differential leukocyte count (DLC) was determined using the Giemsa technique (16, 17). The standard slide-to-slide technique was used to prepare a pair of smears for each blood sample using about 2 μl of blood on a clean microscope slide. The slide was then labeled, air dried, and fixed for 3 min in methanol followed by another air-drying brief period. The slides were then flooded with Wright–Giemsa stain for 3 min followed by addition of an equal amount of Sørensen's buffer (pH 6.8) and gently mixed until the formation of green metallic sheen is seen on the surface. This was followed by washing the stained slides with the Sørensen's buffer, using tissue paper to wipe off the excess stain from the back of the slides and then air dried. The blood smears were examined using a light microscope (Olympus XSZ-107BN) under high-power magnification with oil immersion (×1,000).

The mean corpuscular hemoglobin (MCH) and mean corpuscular hemoglobin concentrations (MCHCs) were calculated as described by Coles (1986).

$$\begin{array}{l} \text{ MCH }(\text{pg})\;=\frac{\text{Hb }(\text{g/dl})\;\times \text{ 10}}{\text{RBC }(\times \;{\text{10}}^{\text{12}}\text{/L})}\\ \text{MCHC }(\text{g/dl})\;=\;\frac{\text{Hb }(\text{g/dl})\;\times \text{ 100}}{\text{PCV }(\%)} \end{array}$$

### Serum Biochemical Analyses

The total protein concentration in each serum sample was determined colorimetrically based on the principle of the Biuret reaction (copper salt in an alkaline medium) by following the manufacturer's instruction (AGAPPE—total protein; AGAPPE Diagnostics Switzerland, GmbH, Cham, Switzerland, 51013002). The absorbance of the standard and sample was measured against the reagent blank using a spectrophotometer (S23A, Techmel, New York, NY, USA) at 546 nm.

The albumin concentration in each serum sample was determined by bromocresol green methodology following the manufacturer's instruction (AGAPPE—albumin; AGAPPE Diagnostics Switzerland, GmbH, 51001002). The sample absorbance was measured against that of reagent blank using a spectrophotometer (S23A, Techmel, USA) at 630 nm.

The globulin concentration in each sample was calculated by subtracting the albumin concentration from the total protein concentration. The albumin–globulin (ALB/GLB) in each sample was calculated by dividing the albumin concentration with globulin concentration.

The urea concentration in each serum sample was determined using the modified Berthelot methodology by following the manufacturer's instruction (AGAPPE—urea B; AGAPPE Diagnostics Switzerland, GmbH, 51216001). The absorbance of the standard and sample against the reagent blank was measured using a spectrophotometer (S23A, Techmel, USA) at 600 nm.

The creatinine concentration in each serum sample was determined using the modified Jaffe's method following the manufacturer's instruction (AGAPPE—creatinine; AGAPPE Diagnostics Switzerland, GmbH, 51009001). The principle is based on the reaction of creatinine with picric acid to produce creatinine alkaline picrate (a colored product). The optical density of mixtures was first taken (T_1_) 60 s after addition of the standard/sample and then again was taken for the second time (T_2_) 60 s after the first reading using a spectrophotometer (S23A, Techmel, USA) at 600 nm.

### Data Analyses

Data were presented using tables and charts. Data were expressed as mean ± SEM and subjected to a two-way analysis of variance (two-way ANOVA) followed by Bonferroni *post-hoc* test (not shown in the tables) using GraphPad Prism version 5.0 (GraphPad, San Diego, CA, USA). Values of *P* ≤ 0.05 were considered significant.

## Results

### Clinical Signs

The incubation period of IBD in pigeons and chickens was 2 days. Anorexia and reduced activity were the clinical signs observed in inoculated pigeons at 2 and 3 dpi, and these signs were absent by 4 dpi. There was no mortality in the pigeons. In inoculated and sentinel chickens, the clinical signs observed included ruffled feathers, anorexia, reduced activity, huddling, somnolence, prostration, and watery diarrhea. Mortalities were 60 and 50% in inoculated and sentinel chickens, respectively.

### Antibody Response

Anti-IBDV antibodies were detected in inoculated and sentinel chickens from 3 dpi, in inoculated pigeons from 4 dpi, and in sentinel pigeons from 5 dpi (Table 1).

**Table 1:** Mean IBDV ELISA titer (at 1:500) of pigeons and chickens following inoculation with and/or exposure to a very virulent infectious bursal disease virus.

	**Uninoculated pigeons**	**Inoculated pigeons**	**Sentinel pigeons**	**Uninoculated chickens**	**Inoculated chickens**	**Sentinel chickens**
**Days postinoculation and/or exposure**	**Mean ELISA titer (at 1:500)**					
0	26.00 ± 12.00	21.67 ± 10.81	24.00 ± 6.00	174.50 ± 14.50	167.00 ± 0.00	162.50 ± 30.50
1	22.00 ± 5.00	16.00 ± 2.00	21.00 ± 3.00	136.00 ± 29.50	213.50 ± 29.50	166.00 ± 4.36
2	13.00 ± 2.08	139.67 ± 7.97	28.00 ± 31.12	92.00 ± 0.00	404.33 ± 39.87	278.67 ± 85.87
3	13.00 ± 1.00	327.33 ± 5.24	223.33 ± 13.02	100.50 ± 8.50	512.33 ± 18.02	530.00 ± 25.00
4	8.00 ± 2.00	557.00 ± 2.00	258.67 ± 29.76	83.67 ± 22.26	544.33 ± 7.84	552.00 ± 11.02
5	11.00 ± 3.00	797.00 ± 40.00	715.00 ± 90.37	87.00 ± 51.05	1006.00 ± 16.00	928.50 ± 153.50
6	2.00 ± 2.00	1245.67 ± 110.64	1000.00 ± 68.00	71.67 ± 32.54	1274.00 ± 56.00	1028.50 ± 16.50
7	0.00 ± 0.00	1463.33 ± 66.16	1337.67 ± 67.77	80.67 ± 35.43	1468 ± 6.50	1545.00 ± 136.00
10	0.00 ± 0.00	1840.50 ± 26.50	1525.00 ± 30.00	60.00 ± 9.00	1932.00 ± 44.00	1822.50 ± 52.50
14	0.00 ± 0.00	2016.33 ± 21.84	1594.00 ± 25.89	43.67 ± 10.17	2143.50 ± 20.50	2175.50 ± 6.50

*Mean ELISA titer >875 indicated positive IBDV antibody detection, while ELISA titer ≤ 875 indicated negative IBDV antibody detection according to the manufacturer's instructions*.

### Changes in Hematology

#### Erythrocytic Parameters

**Figure 1:**
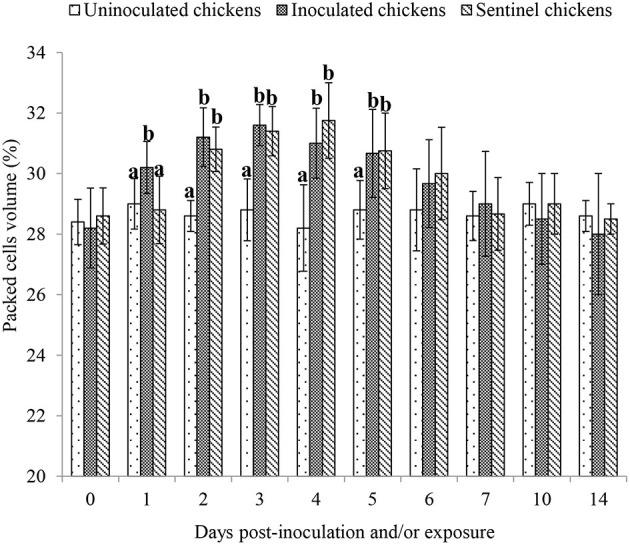
Mean packed cell volume of chickens following inoculation with and/or exposure to a very virulent infectious bursal disease virus. Values with different alphabets in the same day differ significantly at *P* < 0.05.

In pigeons, the packed cell volume (PCV) showed no statistical (*P* > 0.05) difference preinoculation, postinoculation, and/or exposure to 14 dpi/dpe. At 1 dpi, the PCV was significantly (*P* < 0.05) higher in the inoculated chickens than both the uninoculated and sentinel chickens, but the latter two groups were not different from each other (*P* > 0.5). The PCVs of both the inoculated and sentinel groups at 2, 3, 4, and 5 dpi/dpe were significantly higher (*P* < 0.05) than the uninoculated group. From 6 to 14 dpi/dpe, the PCV showed no significant difference (*P* > 0.05) between the treatment groups (Figure 1).

The MCH showed no significant difference (*P* > 0.05) between the treatment groups of both pigeons and chickens except one time point in the chickens. At 3 dpi/dpe, the average MCH of the inoculated group of chickens was significantly lower (*P* < 0.05) than both the uninoculated and sentinel groups (Table 2).

**Table 2:** Mean corpuscular hemoglobin of pigeons and chickens following inoculation with and/or exposure to a very virulent infectious bursal disease virus.

	**Uninoculated pigeons**	**Inoculated pigeons**	**Sentinel pigeons**	**Uninoculated chickens**	**Inoculated chickens**	**Sentinel chickens**
**Days postinoculation and/or exposure**	**Mean corpuscular hemoglobin (pg)**					
0	37.24 ± 3.98^a^	38.59 ± 3.87^a^	38.53 ± 2.02^a^	29.48 ± 0.73^a^	29.52 ± 0.38^a^	29.90 ± 0.01^a^
1	36.39 ± 2.92^a^	38.60 ± 1.95^a^	38.35 ± 2.14^a^	29.72 ± 0.48^a^	28.38 ± 1.53^a^	29.89 ± 0.01^a^
2	37.42 ± 3.33^a^	37.67 ± 2.03^a^	37.93 ± 0.61^a^	29.54 ± 0.23^a^	27.78 ± 1.41^a^	28.46 ± 1.47^a^
3	37.61 ± 2.93^a^	37.47 ± 1.03^a^	38.18 ± 1.42^a^	29.53 ± 0.90^a^	27.05 ± 1.64^b^	27.79 ± 1.39^a^
4	37.29 ± 1.14^a^	37.51 ± 1.72^a^	37.52 ± 1.91^a^	29.20 ± 2.17^a^	26.31 ± 1.86^a^	27.69 ± 1.69^a^
5	37.94 ± 1.43^a^	37.89 ± 1.27^a^	38.54 ± 1.02^a^	29.78 ± 0.13^a^	27.36 ± 2.37^a^	28.77 ± 0.90^b^
6	37.65 ± 1.98^a^	38.24 ± 1.50^a^	37.98 ± 1.56^a^	29.55 ± 1.29^a^	29.06 ± 0.71^a^	29.19 ± 0.37^a^
7	37.70 ± 0.82^a^	37.99 ± 3.28^a^	37.70 ± 0.82^a^	29.70 ± 0.05^a^	29.74 ± 0.02^a^	29.90 ± 0.02^a^
10	37.79 ± 2.24^a^	38.86 ± 4.25^a^	38.64 ± 3.23^a^	29.73 ± 0.02^a^	29.77 ± 0.01^a^	29.93 ± 0.01^a^
14	36.62 ± 1.16^a^	37.25 ± 2.00^a^	36.29 ± 1.18^a^	29.11 ± 0.69^a^	29.20 ± 1.06^a^	29.15 ± 0.81^a^

*Values with different superscript alphabets in the same row for the same species of bird differ significantly at P < 0.05*.

No significant difference was observed in MCHC between the treatment groups of pigeons from 0 to 14 dpi/dpe. The MCHC of the inoculated group of chickens was significantly lower (*P* < 0.05) than that of both the sentinel and uninoculated groups of chickens at 2 dpi/dpe, and the MCHCs of both the inoculated and sentinel groups were significantly lower (*P* < 0.05) than their uninoculated groups at 3 and 4 dpi/dpe (Table 3).

**Table 3:** Mean corpuscular hemoglobin concentration of pigeons and chickens following inoculation with and/or exposure to a very virulent infectious bursal disease virus.

	**Uninoculated pigeons**	**Inoculated pigeons**	**Sentinel pigeons**	**Uninoculated chickens**	**Inoculated chickens**	**Sentinel chickens**
**Days post-inoculation and/or exposure**	**Mean corpuscular hemoglobin concentration (g/dl)**					
0	28.80 ± 2.55^a^	29.59 ± 1.71^a^	29.67 ± 1.00^a^	27.25 ± 0.00^a^	27.24 ± 0.01^a^	27.24 ± 0.01^a^
1	28.18 ± 2.17^a^	29.33 ± 1.57^a^	29.53 ± 1.65^a^	27.25 ± 0.01^a^	26.03 ± 1.21^a^	27.25 ± 0.00^a^
2	29.12 ± 1.44^a^	29.03 ± 2.48^a^	29.40 ± 1.27^a^	27.10 ± 0.00^a^	25.34 ± 1.28^b^	25.93 ± 1.34^a^
3	29.63 ± 1.69^a^	29.42 ± 1.43^a^	29.21 ± 1.46^a^	27.11 ± 0.00^a^	24.67 ± 1.49^b^	25.32 ± 1.26^b^
4	29.07 ± 1.60^a^	29.14 ± 1.53^a^	28.89 ± 1.46^a^	27.10 ± 0.01^a^	23.99 ± 1.71^b^	25.25 ± 1.52^b^
5	29.67 ± 1.00^a^	29.26 ± 1.09^a^	29.67 ± 1.00^a^	27.10 ± 0.01^a^	24.95 ± 2.15^a^	26.22 ± 0.83^a^
6	29.08 ± 1.36^a^	29.67 ± 1.00^a^	29.39 ± 1.20^a^	27.11 ± 0.01^a^	26.48 ± 0.63^a^	26.62 ± 0.32^a^
7	29.14 ± 1.44^a^	28.68 ± 1.78^a^	29.03 ± 0.62^a^	27.11 ± 0.00^a^	27.10 ± 0.01^a^	27.26 ± 0.00^a^
10	28.30 ± 1.59^a^	28.21 ± 1.46^a^	28.65 ± 0.63^a^	27.10 ± 0.00^a^	27.11 ± 0.01^a^	27.24 ± 0.01^a^
14	28.35 ± 1.27^a^	29.12 ± 1.24^a^	28.34 ± 1.05^a^	27.14 ± 0.80^a^	27.17 ± 4.14^a^	27.19 ± 0.64^a^

*Values with different superscript alphabets in the same row for the same species of bird differ significantly at P < 0.05*.

#### Leukocytic Parameters

At 3 dpi/dpe, total leukocyte count (TLC) was significantly (*P* < 0.05) increased in the inoculated group of pigeons compared to the uninoculated and sentinel groups of pigeons. The TLC was observed significantly lower (*P* < 0.05) in the inoculated group of chickens than its counterparts of the sentinel and uninoculated groups at 1 dpi/dpe, and both the inoculated and sentinel groups had significantly lower (*P* < 0.05) TLC than the uninoculated group of chickens at 2 dpi/dpe. From 3 to 14 dpi/dpe, however, the observed TLC of the inoculated and sentinel groups of chickens were significantly higher (*P* < 0.05) than their counterpart of the uninoculated groups. The TLC of both the inoculated and the sentinel groups of chickens reached a peak at 5 dpi/dpe, followed with gradual decreases (Table 4).

**Table 4:** Mean total leukocyte count of pigeons and chickens following inoculation with and/or exposure to a very virulent infectious bursal disease virus.

	**Uninoculated pigeons**	**Inoculated pigeons**	**Sentinel pigeons**	**Uninoculated chickens**	**Inoculated chickens**	**Sentinel chickens**
**Days postinoculation and/or exposure**	**Total leukocytes count (×10**^****9****^**/l)**					
0	22.20 ± 1.18^a^	21.90 ± 1.13^a^	21.66 ± 1.19^a^	25.68 ± 0.66^a^	25.96 ± 1.53^a^	25.98 ± 0.89^a^
1	21.88 ± 0.52^a^	21.68 ± 0.78^a^	21.62 ± 1.10^a^	25.62 ± 1.01^a^	22.42 ± 1.05^b^	25.72 ± 1.02^a^
2	22.12 ± 0.91^a^	20.92 ± 1.29^a^	21.10 ± 1.47^a^	25.96 ± 1.03^a^	23.02 ± 0.76^b^	23.48 ± 0.80^b^
3	21.90 ± 0.95^a^	23.86 ± 1.71^b^	22.02 ± 1.51^a^	25.52 ± 1.04^a^	28.64 ± 1.06^b^	29.22 ± 1.45^b^
4	21.80 ± 0.99^a^	21.42 ± 1.20^a^	21.58 ± 1.29^a^	25.50 ± 0.58^a^	36.07 ± 1.67^b^	37.63 ± 1.50^b^
5	21.56 ± 1.01^a^	21.90 ± 1.43^a^	20.68 ± 1.50^a^	26.18 ± 1.02^a^	48.43 ± 2.48^b^	46.30 ± 3.04^b^
6	21.76 ± 0.90^a^	21.70 ± 1.11^a^	20.72 ± 1.01^a^	25.96 ± 1.56^a^	40.80 ± 1.23^b^	38.83 ± 3.64^b^
7	22.06 ± 1.26^a^	21.26 ± 1.04^a^	20.72 ± 1.03^a^	26.38 ± 1.75^a^	32.77 ± 1.24^b^	31.13 ± 1.96^b^
10	21.84 ± 0.90^a^	21.36 ± 1.23^a^	21.58 ± 0.99^a^	26.60 ± 1.16^a^	29.35 ± 1.65^b^	29.00 ± 0.80^b^
14	21.88 ± 0.61^a^	21.52 ± 0.78^a^	21.80 ± 0.64^a^	26.28 ± 1.76^a^	28.20 ± 1.70^b^	28.35 ± 1.65^b^

*Values with different superscript alphabets in the same row for the same species of bird differ significantly at P < 0.05*.

The heterophil counts of pigeons and chickens pre- and postinoculation/exposure to IBDV are presented in Table 5. There was a significant (*P* < 0.05) increase in heterophil count of inoculated pigeons at 3 dpi compared to the uninoculated and sentinel pigeons. In chickens, from 3 to 14 dpi/dpe, heterophil counts of both the inoculated and sentinel groups of chickens were significantly higher (*P* < 0.05) than the uninoculated control groups. The heterophil count reached the highest at 5 dpi/dpe in the inoculated and sentinel chickens and then gradually declined all the way to the 14 dpi/dpe.

**Table 5:** Mean heterophil count of pigeons and chickens following inoculation with and/or exposure to a very virulent infectious bursal disease virus.

	**Uninoculated pigeons**	**Inoculated pigeons**	**Sentinel pigeons**	**Uninoculated chickens**	**Inoculated chickens**	**Sentinel chickens**
**Days postinoculation and/or exposure**	**Heterophil count (×10**^****9****^**/l)**					
0	7.70 ± 0.29^a^	7.42 ± 0.66^a^	7.84 ± 0.84^a^	9.99 ± 0.23^a^	9.38 ± 0.78^a^	9.37 ± 0.89^a^
1	8.01 ± 0.67^a^	7.94 ± 0.19^a^	8.10 ± 0.46^a^	9.99 ± 0.50^a^	9.11 ± 0.60^a^	9.80 ± 1.02^a^
2	7.94 ± 0.29^a^	8.06 ± 0.60^a^	8.02 ± 0.43^a^	8.98 ± 0.32^a^	9.30 ± 0.34^a^	9.47 ± 0.80^a^
3	7.68 ± 0.52^a^	8.40 ± 0.73^b^	7.96 ± 0.69^a^	8.59 ± 0.47^a^	12.43 ± 0.71^b^	11.20 ± 1.45^b^
4	7.60 ± 0.37^a^	7.58 ± 0.38^a^	7.44 ± 0.73^a^	9.22 ± 0.40^a^	17.00 ± 1.57^b^	17.88 ± 1.50^b^
5	7.62 ± 0.71^a^	7.72 ± 0.60^a^	7.52 ± 0.72^a^	8.58 ± 0.72^a^	28.02 ± 0.70^b^	25.98 ± 3.04^b^
6	7.42 ± 0.84^a^	7.58 ± 0.60^a^	7.42 ± 0.54^a^	8.70 ± 0.85^a^	23.02 ± 1.61^b^	23.44 ± 3.64^b^
7	7.54 ± 0.35^a^	7.66 ± 0.60^a^	7.56 ± 0.46^a^	9.23 ± 0.72^a^	14.24 ± 1.11^b^	15.97 ± 1.96^b^
10	7.66 ± 0.57^a^	7.58 ± 0.62^a^	7.68 ± 0.32^a^	8.57 ± 0.45^a^	11.69 ± 0.40^b^	11.96 ± 1.30^b^
14	7.32 ± 0.54^a^	7.64 ± 0.41^a^	7.52 ± 0.47^a^	8.95 ± 1.29^a^	11.42 ± 0.35^b^	11.48 ± 2.15^b^

*Values with different superscript alphabets in the same row for the same species of bird differ significantly at P < 0.05*.

The lymphocyte counts of pigeons and chickens following inoculation and/or exposure to IBDV are presented in Table 6. No difference in lymphocyte count was observed between the treatment groups of pigeons (*P* > 0.05). The observed lymphocyte counts were significantly lower in the inoculated groups of chickens at 1–4 dpi and in the sentinel groups at 2–4 dpe (*P* < 0.05). No significant difference in lymphocyte count was observed between the treatment groups of chickens from 5 to 14 dpi/dpe.

**Table 6:** Mean lymphocyte count of pigeons and chickens following inoculation with and/or exposure to a very virulent infectious bursal disease virus.

	**Uninoculated pigeons**	**Inoculated pigeons**	**Sentinel pigeons**	**Uninoculated chickens**	**Inoculated chickens**	**Sentinel chickens**
**Days postinoculation and/or exposure**	**Lymphocyte count (×10**^****9****^**/l)**					
	13.38 ± 0.78^a^	13.24 ± 0.50^a^	13.36 ± 0.77^a^	15.82 ± 0.60^a^	16.38 ± 0.97^a^	16.35 ± 0.51^a^
1	12.96 ± 0.74^a^	13.04 ± 0.90^a^	13.34 ± 0.73^a^	15.98 ± 0.90^a^	13.03 ± 0.73^b^	15.99 ± 0.73^a^
2	13.22 ± 0.86^a^	13.18 ± 1.50^a^	13.14 ± 1.04a	16.78 ± 1.14^a^	13.20 ± 0.42^b^	13.48 ± 0.53^b^
3	13.30 ± 0.65^a^	12.98 ± 0.65^a^	13.04 ± 1.40^a^	16.63 ± 0.65^a^	14.34 ± 0.45^b^	14.89 ± 0.69^b^
4	13.30 ± 0.90^a^	12.92 ± 0.75^a^	13.26 ± 0.69^a^	16.43 ± 0.66^a^	14.53 ± 0.68^b^	15.35 ± 0.82^b^
5	13.24 ± 0.50^a^	13.14 ± 1.08^a^	13.18 ± 0.65^a^	17.15 ± 1.16^a^	15.84 ± 0.76^a^	16.19 ± 0.69^a^
6	13.16 ± 0.68^a^	12.94 ± 0.57^a^	12.98 ± 0.57^a^	17.01 ± 1.35^a^	17.23 ± 1.00^a^	16.86 ± 0.61^a^
7	13.38 ± 0.92^a^	13.16 ± 0.43^a^	13.30 ± 0.58^a^	16.89 ± 1.39^a^	17.13 ± 0.26^a^	17.21 ± 0.71^a^
10	13.28 ± 0.62^a^	12.98 ± 0.49^a^	12.98 ± 0.97^a^	17.04 ± 1.04^a^	17.22 ± 0.31^a^	17.13 ± 0.23^a^
14	13.30 ± 0.42^a^	13.00 ± 0.49^a^	13.10 ± 0.48^a^	17.19 ± 1.04^a^	17.16 ± 1.08^a^	16.79 ± 1.00^a^

*Values with different superscript alphabets in the same row for the same species of bird differ significantly at P < 0.05*.

Heterophil/lymphocyte ratios (H/L) were calculated. IBDV infection significantly upregulated the H/L in pigeons only at 3 dpi (*P* < 0.05). Significantly higher H/L (*P* < 0.05) were observed in the inoculated groups of chickens from 1 to 14 dpi and in the sentinel groups of chickens from 2 to 14 dpe than the uninoculated control group of chickens (Table 7).

**Table 7:** Mean heterophil/lymphocyte ratio of pigeons and chickens following inoculation with and/or exposure to a very virulent infectious bursal disease virus.

	**Uninoculated pigeons**	**Inoculated pigeons**	**Sentinel pigeons**	**Uninoculated chickens**	**Inoculated chickens**	**Sentinel chickens**
**Days postinoculation and/or exposure**	**Heterophil/lymphocyte ratio**					
0	0.55 ± 0.01^a^	0.54 ± 0.04^a^	0.52 ± 0.04^a^	0.63 ± 0.03^a^	0.57 ± 0.04^a^	0.57 ± 0.03^a^
1	0.53 ± 0.04^a^	0.57 ± 0.03^a^	0.55 ± 0.04^a^	0.63 ± 0.05^a^	0.70 ± 0.03^b^	0.62 ± 0.03^a^
2	0.55 ± 0.03^a^	0.57 ± 0.04^a^	0.56 ± 0.06^a^	0.55 ± 0.04^a^	0.70 ± 0.01^b^	0.70 ± 0.01^b^
3	0.51 ± 0.05^a^	0.60 ± 0.06^b^	0.53 ± 0.07^ab^	0.52 ± 0.01^a^	0.87 ± 0.04^b^	0.76 ± 0.06^b^
4	0.53 ± 0.04^a^	0.57 ± 0.04^a^	0.56 ± 0.05^a^	0.58 ± 0.04^a^	1.17 ± 0.05^b^	1.14 ± 0.07^b^
5	0.54 ± 0.03^a^	0.56 ± 0.08^a^	0.52 ± 0.05^a^	0.51 ± 0.06^a^	1.67 ± 0.06^b^	1.59 ± 0.14^b^
6	0.56 ± 0.07^a^	0.58 ± 0.05^a^	0.57 ± 0.03^a^	0.53 ± 0.07^a^	1.35 ± 0.17^b^	1.34 ± 0.06^b^
7	0.56 ± 0.04^a^	0.58 ± 0.05^a^	0.57 ± 0.04^a^	0.56 ± 0.07^a^	0.82 ± 0.07^b^	0.81 ± 0.10^b^
10	0.55 ± 0.04^a^	0.57 ± 0.04^a^	0.56 ± 0.02^a^	0.51 ± 0.02^a^	0.68 ± 0.04^b^	0.66 ± 0.06^b^
14	0.50 ± 0.04^a^	0.53 ± 0.04^a^	0.52 ± 0.03^a^	0.52 ± 0.08^a^	0.67 ± 0.06^b^	0.65 ± 0.05^b^

*Values with different superscript alphabets in the same row for the same species of bird differ significantly at P < 0.05*.

### Changes in Serum Biochemistry

#### Total Protein, Globulin Concentrations, and Albumin/Globulin Ratio

The average serum total protein concentration was significantly upregulated in the treatment groups of pigeons in response to IBDV inoculation or exposure at 10 and 14 dpi/dpe in contrast to the uninoculated control groups (*P* < 0.05). The significantly upregulated total protein concentration was also observed (*P* < 0.05) in inoculated and sentinel groups of chickens at 3, 4, and 7–14 dpi/dpe in comparison with the counterpart of uninoculated control groups (Table 8).

**Table 8:** Mean serum total protein concentration of pigeons and chickens following inoculation with and/or exposure to a very virulent infectious bursal disease virus.

	**Uninoculated pigeons**	**Inoculated pigeons**	**Sentinel pigeons**	**Uninoculated chickens**	**Inoculated chickens**	**Sentinel chickens**
**Days postinoculation and/or exposure**	**Serum total protein concentration (g/dl)**					
0	4.05 ± 0.25^a^	4.05 ± 0.47^a^	4.08 ± 0.37^a^	3.28 ± 0.09^a^	3.30 ± 0.12^a^	3.28 ± 0.11^a^
1	4.25 ± 0.23^a^	4.10 ± 0.15^a^	4.13 ± 0.11^a^	3.35 ± 0.30^a^	3.40 ± 0.16^a^	3.28 ± 0.11^a^
2	4.03 ± 0.40^a^	4.03 ± 0.20^a^	4.10 ± 0.41^a^	3.38 ± 0.06^a^	3.43 ± 0.14^a^	3.43 ± 0.17^a^
3	4.20 ± 0.27^a^	4.05 ± 0.06^a^	4.08 ± 0.32^a^	3.35 ± 0.09^a^	3.60 ± 0.15^b^	3.57 ± 0.20^b^
4	4.10 ± 0.45^a^	4.10 ± 0.27^a^	4.13 ± 0.38^a^	3.40 ± 0.15^a^	3.65 ± 0.05^b^	3.63 ± 0.12^b^
5	4.28 ± 0.40^a^	4.13 ± 0.18^a^	4.13 ± 0.41^a^	3.50 ± 0.20^a^	3.55 ± 0.25^a^	3.47 ± 0.35^a^
6	4.10 ± 0.34^a^	4.35 ± 0.30^a^	4.33 ± 0.39^a^	3.50 ± 0.20^a^	3.55 ± 0.35^a^	3.53 ± 0.27^a^
7	4.30 ± 0.31^a^	4.50 ± 0.51^a^	4.48 ± 0.23^a^	3.35 ± 0.09^a^	3.50 ± 0.10^b^	3.53 ± 0.15^b^
10	4.18 ± 0.29^a^	4.75 ± 0.16^b^	4.70 ± 0.29^b^	3.38 ± 0.09^a^	3.60 ± 0.10^b^	3.65 ± 0.15^b^
14	4.33 ± 0.30^a^	4.85 ± 0.23^b^	4.78 ± 0.24^b^	3.48 ± 0.20^a^	3.75 ± 0.15^b^	3.80 ± 0.20^b^

*Values with different superscript alphabets in the same row for the same species of bird differ significantly at P < 0.05*.

The average serum globulin concentration of the IBDV inoculated and sentinel groups of both pigeons and chickens was significantly higher (*P* < 0.05) at 10 and 14 dpi/dpe than the uninoculated control groups (Table 9). The average albumin–globulin ratios (A/G) of both the inoculated and sentinel groups of pigeons and chickens were significantly lower (*P* < 0.05) than their uninoculated control groups at 10 and 14 dpi/dpe (Table 10).

**Table 9:** Mean serum globulin concentration of pigeons and chickens following inoculation with and/or exposure to a very virulent infectious bursal disease virus.

	**Uninoculated pigeons**	**Inoculated pigeons**	**Sentinel pigeons**	**Uninoculated chickens**	**Inoculated chickens**	**Sentinel chickens**
**Days postinoculation and/or exposure**	**Serum globulin concentration (g/dl)**					
0	2.01 ± 0.30^a^	2.03 ± 0.50^a^	2.04 ± 0.30^a^	1.83 ± 0.08^a^	1.87 ± 0.08^a^	1.84 ± 0.18^a^
1	2.18 ± 0.15^a^	2.10 ± 0.22^a^	2.17 ± 0.25^a^	1.86 ± 0.32^a^	1.82 ± 0.24^a^	1.76 ± 0.16^a^
2	2.05 ± 0.22^a^	1.94 ± 0.11^a^	2.14 ± 0.40^a^	1.81 ± 0.08^a^	1.79 ± 0.22^a^	1.78 ± 0.25^a^
3	2.12 ± 0.28^a^	2.18 ± 0.19^a^	2.19 ± 0.50^a^	1.84 ± 0.15^a^	2.05 ± 0.19^a^	2.01 ± 0.08^a^
4	2.06 ± 0.35^a^	2.16 ± 0.23^a^	2.09 ± 0.60^a^	1.90 ± 0.16^a^	2.08 ± 0.03^a^	2.06 ± 0.25^a^
5	2.00 ± 0.29^a^	2.06 ± 0.31^a^	2.10 ± 0.45^a^	1.90 ± 0.18^a^	1.89 ± 0.46^a^	1.77 ± 0.33^a^
6	2.10 ± 0.34^a^	2.29 ± 0.35^a^	2.35 ± 0.47^a^	1.87 ± 0.33^a^	1.97 ± 0.00^a^	1.94 ± 0.40^a^
7	2.11 ± 0.32^a^	2.54 ± 0.24^a^	2.56 ± 0.36^a^	1.82 ± 0.12^a^	2.01 ± 0.08^a^	2.02 ± 0.26^a^
10	2.18 ± 0.35^a^	2.68 ± 0.04^b^	2.73 ± 0.14^b^	1.77 ± 0.14^a^	2.17 ± 0.04^b^	2.21 ± 0.23^b^
14	2.21 ± 0.16^a^	2.74 ± 0.24^b^	2.65 ± 0.26^b^	1.84 ± 0.26^a^	2.25 ± 0.12^b^	2.24 ± 0.09^b^

*Values with different superscript alphabets in the same row for the same species of bird differ significantly at P < 0.05*.

**Table 10:** Mean albumin/globulin ratio of pigeons and chickens following inoculation with and/or exposure to a very virulent infectious bursal disease virus.

	**Uninoculated pigeons**	**Inoculated pigeons**	**Sentinel pigeons**	**Uninoculated chickens**	**Inoculated chickens**	**Sentinel chickens**
**Days postinoculation and/or exposure**	**Albumin/globulin ratio**					
0	1.12 ± 0.25^a^	1.05 ± 0.24^a^	1.10 ± 0.30^a^	0.79 ± 0.03^a^	0.77 ± 0.02^a^	0.79 ± 0.12^a^
1	1.08 ± 0.14^a^	1.02 ± 0.19^a^	0.98 ± 0.24^a^	0.82 ± 0.13^a^	0.86 ± 0.12^a^	0.85 ± 0.10^a^
2	1.09 ± 0.17^a^	1.08 ± 0.06^a^	1.12 ± 0.35^a^	0.87 ± 0.10^a^	0.88 ± 0.12^a^	0.87 ± 0.11^a^
3	1.14 ± 0.21^a^	1.14 ± 0.14^a^	1.14 ± 0.43^a^	0.85 ± 0.12^a^	0.78 ± 0.13^a^	0.77 ± 0.05^a^
4	1.05 ± 0.13^a^	0.93 ± 0.11^a^	0.97 ± 0.12^a^	0.81 ± 0.07^a^	0.76 ± 0.05^a^	0.79 ± 0.12^a^
5	1.05 ± 0.23^a^	1.11 ± 0.23^a^	1.01 ± 0.31^a^	0.86 ± 0.07^a^	0.86 ± 0.25^a^	0.85 ± 0.10^a^
6	1.04 ± 0.21^a^	0.97 ± 0.16^a^	1.09 ± 0.43^a^	0.86 ± 0.11^a^	0.81 ± 0.18^a^	0.82 ± 0.20^a^
7	0.95 ± 0.27^a^	0.87 ± 0.23^a^	0.93 ± 0.13^a^	0.85 ± 0.08^a^	0.74 ± 0.11^a^	0.75 ± 0.15^a^
10	1.13 ± 0.32^a^	0.77 ± 0.04^b^	0.78 ± 0.14^b^	0.94 ± 0.12^a^	0.66 ± 0.08^b^	0.64 ± 0.11^b^
14	1.06 ± 0.17^a^	0.82 ± 0.13^b^	0.83 ± 0.12^b^	0.92 ± 0.15^a^	0.60 ± 0.08^b^	0.65 ± 0.12^b^

*Values with different superscript alphabets in the same row for the same species of bird differ significantly at P < 0.05*.

#### Urea and Creatinine Concentrations

The average serum urea concentration was significantly higher (*P* < 0.05) in the IBDV inoculated (1.01 ± 0.05 mg/dl, 1.02 ± 0.01 mg/dl, 0.92 ± 0.04 mg/dl) and sentinel (0.97 ± 0.05 mg/dl, 0.99 ± 0.07 mg/dl, 0.85 ± 0.08 mg/dl) groups of chickens than the corresponding uninoculated control groups at 3, 4, and 5 dpi/dpe (respectively). No significant difference was observed between the treatment groups of chickens at the rest of dpi/dpe, and no difference was observed between the treatment groups of pigeons at any dpi/dpe (Table 11). No significant difference was observed in average serum creatinine concentration between the treatment groups of either pigeons or chickens in response to the IBDV inoculation or exposure during the experimental period (Table 12).

**Table 11:** Mean serum urea concentration of pigeons and chickens following inoculation with and/or exposure to a very virulent infectious bursal disease virus.

	**Uninoculated pigeons**	**Inoculated pigeons**	**Sentinel pigeons**	**Uninoculated chickens**	**Inoculated chickens**	**Sentinel chickens**
**Days postinoculation and/or exposure**	**Serum urea concentration (mg/dl)**					
0	0.30 ± 0.06^a^	0.30 ± 0.05^a^	0.29 ± 0.06^a^	0.86 ± 0.06^a^	0.89 ± 0.11^a^	0.90 ± 0.04^a^
1	0.31 ± 0.03^a^	0.32 ± 0.06^a^	0.30 ± 0.04^a^	0.88 ± 0.05^a^	0.89 ± 0.10^a^	0.85 ± 0.06^a^
2	0.32 ± 0.09^a^	0.34 ± 0.07^a^	0.34 ± 0.09^a^	0.85 ± 0.03^a^	0.92 ± 0.07^a^	0.88 ± 0.01^a^
3	0.34 ± 0.08^a^	0.36 ± 0.08^a^	0.33 ± 0.08^a^	0.86 ± 0.06^a^	1.01 ± 0.05^b^	0.97 ± 0.05^b^
4	0.31 ± 0.05^a^	0.33 ± 0.04^a^	0.32 ± 0.12^a^	0.81 ± 0.05^a^	1.02 ± 0.01^b^	0.99 ± 0.07^b^
5	0.32 ± 0.06^a^	0.29 ± 0.06^a^	0.34 ± 0.05^a^	0.76 ± 0.05^a^	0.92 ± 0.04^b^	0.85 ± 0.08^ab^
6	0.32 ± 0.07^a^	0.30 ± 0.04^a^	0.31 ± 0.03^a^	0.81 ± 0.11^a^	0.82 ± 0.03^a^	0.81 ± 0.09^a^
7	0.34 ± 0.07^a^	0.34 ± 0.09^a^	0.34 ± 0.05^a^	0.83 ± 0.05^a^	0.85 ± 0.07^a^	0.84 ± 0.04^a^
10	0.33 ± 0.06^a^	0.33 ± 0.08^a^	0.33 ± 0.10^a^	0.82 ± 0.08^a^	0.85 ± 0.10^a^	0.86 ± 0.03^a^
14	0.33 ± 0.04^a^	0.32 ± 0.12^a^	0.32 ± 0.06^a^	0.81 ± 0.11^a^	0.84 ± 0.03^a^	0.83 ± 0.02^a^

*Values with different alphabets in the same row for the same species differ significantly at P < 0.05*.

**Table 12:** Mean serum creatinine concentration of pigeons and chickens following inoculation with and/or exposure to a very virulent infectious bursal disease virus.

	**Uninoculated pigeons**	**Inoculated pigeons**	**Sentinel pigeons**	**Uninoculated chickens**	**Inoculated chickens**	**Sentinel chickens**
**Days postinoculation and/or exposure**	**Serum creatinine concentration (mg/dl)**					
0	0.43 ± 0.08	0.42 ± 0.07	0.43 ± 0.08	0.43 ± 0.02	0.44 ± 0.02	0.43 ± 0.03
1	0.46 ± 0.05	0.47 ± 0.07	0.46 ± 0.04	0.44 ± 0.02	0.44 ± 0.04	0.43 ± 0.02
2	0.46 ± 0.11	0.49 ± 0.10	0.49 ± 0.13	0.46 ± 0.02	0.46 ± 0.02	0.45 ± 0.02
3	0.49 ± 0.11	0.52 ± 0.11	0.49 ± 0.10	0.45 ± 0.02	0.43 ± 0.03	0.44 ± 0.03
4	0.44 ± 0.07	0.47 ± 0.05	0.46 ± 0.17	0.47 ± 0.02	0.44 ± 0.05	0.45 ± 0.02
5	0.46 ± 0.09	0.47 ± 0.06	0.48 ± 0.06	0.46 ± 0.02	0.48 ± 0.00	0.46 ± 0.03
6	0.45 ± 0.09	0.45 ± 0.04	0.45 ± 0.03	0.45 ± 0.02	0.44 ± 0.00	0.45 ± 0.03
7	0.49 ± 0.10	0.49 ± 0.13	0.49 ± 0.05	0.48 ± 0.01	0.47 ± 0.02	0.48 ± 0.01
10	0.47 ± 0.09	0.49 ± 0.11	0.48 ± 0.13	0.47 ± 0.03	0.50 ± 0.02	0.49 ± 0.04
14	0.47 ± 0.06	0.46 ± 0.17	0.47 ± 0.08	0.44 ± 0.01	0.46 ± 0.05	0.44 ± 0.01

## Discussion

The detection of anti-IBDV antibodies in pigeons and chickens in this study indicated serological evidence of IBDV infection. There was no significant alterations in PCV, MCH, and MCHC of inoculated and sentinel pigeons. In inoculated and sentinel chickens, there was significant polycythemia and decreased MCH and MCHC. However, the polycythemia was relative due to insignificant alterations in the red blood cell (RBC). Polycythemia following IBDV infection in chickens has also been reported by Oladele et al. (12), Bello et al. (13), and Chineme and Cho (18). In contrast, anemia due to IBDV infection has been documented in turkeys and guinea fowls (12). The relative polycythemia observed in inoculated and sentinel chickens might be associated with hemoconcentration due to dehydration resulting from anorexia and diarrhea (12, 13). The decrease in MCH and MCHC in this study suggested normocytic hypochromic anemia, and this might have resulted from hemorrhage with subsequent iron deficiency. Thus, the type of anemia encountered in vvIBDV infection in chickens in this study could be considered as normocytic hypochromic anemia.

Leukocytosis due to heterophilia in vvIBDV-inoculated pigeons was short lived and absent in sentinel pigeons. In the chickens, leukocytic response was characterized by initial leukopenia due to lymphopenia followed by leukocytosis due to heterophilia. Since heterophils have been known to play critical role in phagocytosis of tissue debris (16), the absence of heterophilic response in sentinel pigeons suggested no tissue destruction. The short-lived heterophilia in inoculated pigeons was suggestive of mild tissue destruction by the vvIBDV. In contrast, the persistence of heterophilia in chickens was indicative of massive tissue destruction (12). Lymphopenia, as observed in this study, has been reported to occur following IBDV infection in chickens, and this was attributed to excess glucocorticoid release (19, 20) and/or specific destruction of lymphocytes by IBDV within the BF (21).

Although heterophil/lymphocyte ratio has been documented to be an indicator of stress in birds, it does not indicate susceptibility or response to disease challenge (20, 22, 23). The transient high heterophil/lymphocyte ratio observed in the inoculated pigeons of this study might be due to the short-lived heterophilic response to vvIBDV infection. Similarly, the persistently high heterophil/lymphocyte ratio observed in the inoculated and sentinel chickens was due to a prolongedly heterophilic response to the IBDV infection. In addition, decreased relative space per bird resulting from competition with the pigeons might have induced further increase in corticosterone levels of the chickens with consequent lymphopenia and heterophilia (20, 24). Species differences and/or differences in susceptibility to vvIBDV might be the possible reason for the variation in erythocytic, heterophilic, and lymphocytic responses of pigeons and chickens observed in this study.

The significant hyperproteinemia, consequent hyperglobulinemia, and decreased albumin/globulin ratio observed in pigeons following infection and/or exposure at 10 and 14 dpi/dpe was possibly due to increase in immunoglobulins in response to vvIBDV infection. In chickens, the initial hyperproteinemia at 3 and 4 dpi/dpe might be due to dehydration (25). This suggestion is justified by the concomitant polycythemia with unaltered erythrocytes count. The later hyperproteinemia and subsequent decreased albumin/globulin ratio might probably have resulted from hyperglobulinemia due to increased immunoglobulins in response to vvIBDV infection and decreased albumin level. However, the absence of significant changes in albumin/globulin ratio at 3 and 4 dpi/dpe in chickens, despite hyperproteinemia, might be due to decreased albumin resulting from hemorrhages and anorexia caused by vvIBDV infection. This is consistent with the report by Lumeij (1987) who suggested possible hyperproteinemia with normal albumin/globulin ratio in dehydrated birds (26). The albumin/globulin ratio has been documented to be a good indicator of health and response to diseases (27, 28); hence, the decreased albumin/globulin ratio in pigeons and chickens postinfection/exposure in this study is indicative of immune response to the vvIBDV infection.

Serum/plasma urea and creatinine concentrations have been suggested to be indicators of the extent of renal damage and muscle destruction, respectively (29). In this study, there were no significant changes in serum urea and creatinine concentrations of inoculated and sentinel pigeons up to 14 dpi/dpe, and this is suggestive of minimal or absence of renal damage and muscle destruction as well as dehydration. The increased serum urea concentrations of inoculated and sentinel chickens might be due to dehydration resulting from anorexia and watery diarrhea caused by the vvIBDV infection (30). Since urea is excreted mainly by glomerular filtration, the rate of tubular reabsorption is dependent on hydration condition such that during dehydration all filtered urea is reabsorbed (31). This constitutes the possible reason for elevated urea concentrations in chickens in this study. However, there were no significant changes in creatinine concentrations of chickens following infection/exposure despite dehydration, and this suggests minimal muscle destruction. Another possible reason is that creatinine excretion occurs mainly by tubular secretion, which is not dependent on rate of urine flow (32) and so was not affected by dehydration.

## Conclusion

The leukocytosis, heterophilia, hyperproteinemia, and hyperglobulinemia demonstrated in pigeons post-vvIBDV infection/exposure in this study suggest that they are susceptible to IBD. To the authors' knowledge, this is the first report on hematological and serum biochemical alterations in pigeons due to vvIBDV infection.

## Data Availability Statement

The original contributions presented in the study are included in the article/supplementary material, further inquiries can be directed to the corresponding author/s.

## Ethics Statement

The animal study was reviewed and approved by Ahmadu Bello University Committee on Animal Use and Care.

## Author Contributions

SO, PA, and KE conceptualized and design the experiment, edited, and review the manuscript. OO, TM, SE, AA, MM, and SU performed the experiment and analyzed the data. OO and TM drafted the manuscript. All authors have read and agreed to the published version of the manuscript.

## Conflict of Interest

The authors declare that the research was conducted in the absence of any commercial or financial relationships that could be construed as a potential conflict of interest.
